# Acute disseminated encephalomyelitis and viral encephalitis: An unusual and misleading imaging

**DOI:** 10.1002/ccr3.8004

**Published:** 2023-09-30

**Authors:** Isha Shukla, Bhavin Modasia, Bipin Chaurasia

**Affiliations:** ^1^ Park Hospital Gurugram Harayana India; ^2^ Department of Neurosurgery Neurosurgery Clinic Birgunj Nepal

**Keywords:** ADEM, demyelination, encephalitis, intravenous immunoglobulins, steroids, symmetrical white and gray matter lesions

## Abstract

Acute disseminated encephalomyelitis (ADEM) is a rare illness. It is characterized by different presentations like encephalopathy, seizures, hemiplegia, and visual symptoms. We present a patient who presented seizures and encephalopathy. Brain MRI showed symmetrical white and gray matter lesions. He was treated with acyclovir for viral encephalitis and given immunotherapy for ADEM. The radiological findings may be inconclusive in some cases, hence differential diagnosis of both viral encephalitis and ADEM needs to be considered. Early immunotherapy is required in such fulminant cases.

## Graphical abstract

Acute hemorrhagic ecephalomyelitis (ADEM).

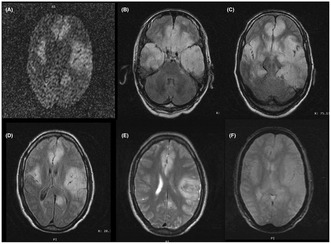

## 1 INTRODUCTION

Acute disseminated encephalomyelitis (ADEM) affects 125,000–250,000 individuals each year.^1^ ADEM is diagnosed on basis of clinical presentations and radiological findings.^2^, ^3^

The history of inciting agent and temporal relationship may or may not be present. It has been seen that 50–75% ADEM cases were preceded by infection or vaccination. Historically measles virus has been shown to induce disease in approximately one per 400–1000 cases,^1^ while ADEM can occur in one per 10,000 varicella vaccination administered. Recently SARS–COV2 infection has been associated with ADEM cases. In 2% of cases, hyperacute or malignant ADEM is seen, which is characterized by rapid symptoms onset followed by catastrophic and massive brain edema and its sequelae.

## 2 CASE DESCRIPTION

A 57‐year‐old male patient presented with generalized seizures occurring in a time of 1 h with no regain of consciousness. He had headache and low‐grade fever for 1 day. No history of seizures. No history of smoking, alcohol intake, or substance abuse.

At admission, patient was disoriented and occasionally obeyed verbal commands. He presented a GCS score of 8 (E2M5V1). He was moving all four limbs and his vitals were stable. Plantar reflex was flexor bilaterally. There was no neck rigidity. Patient deteriorated on second day, with a GCS score of 5 (E2M2V1). Hence, he was intubated and connected to mechanical ventilator.

**FIGURE 1:**
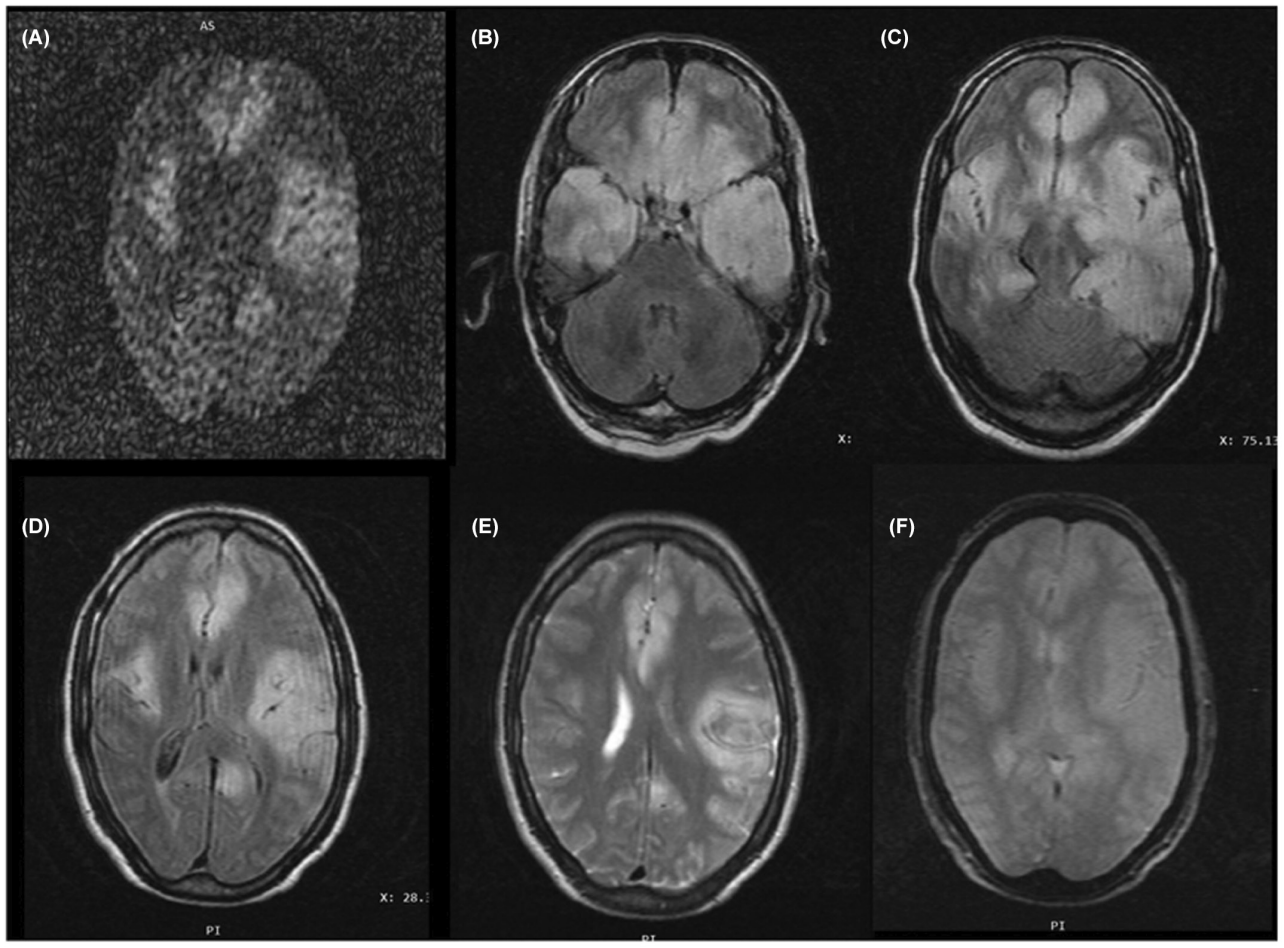
Brain MRI showed abnormal signal, T2/FLAIR (fluid attenuated inversion recovery) sequence showed hyperintensities, T1 sequence showed hypo‐intensities with symmetrical involvement of bilateral medial temporal lobes, bilateral external capsule, basal ganglia, subcortical part of frontal lobes and basifrontal lobes. GRE (Gradient echo) sequences did not show hemorrhages, hence acute hemorrhagic encephalomyelitis which is a form of ADEM was ruled out (A–F).

Patient was evaluated for malaria, dengue, typhoid, and scrub typhus which are the region‐specific causes of fever and altered sensorium: tests were negative for above. Cerebrospinal fluid (CSF) examination showed glucose 97 mg/dL (Reference 40–80 mg/dL), protein 239 mg/dL (Reference 15–45 mg/dL), total leukocyte count was 80/μL (Reference 0–5/μL), 70% lymphocytes, 30% polymorphonuclear cells. Blood sugar was 113 mg/dL at time of lumbar puncture. Herpes PCR and other neurotropic virus panel was negative. CSF acid fast bacilli and cartridge‐based nucleic acid amplification test (CBNAAT) for Mycobacterium tuberculosis was negative. CSF bacterial cultures and fungal study were also negative. CSF oligoclonal band was negative. Myelin oligo dendrocyte glycoprotein (MOG) antibodies were negative in serum. Autoimmune encephalitis panel, antibodies against following antigens: N‐methyl‐d‐aspartate (NMDA) anti‐glutamate receptor against NR1 subunit, Anti‐glutamate receptor (AMPA‐GluR1), alpha‐amino‐3‐hydroxy‐5‐methyl‐4‐isoxazol‐propionic acid (AMPA‐GluR2), gamma amino butyric acid B receptor (GABA‐B), leucine‐rich glioma‐inactivated protein 1 (LGi‐1), contactin‐associated protein 2 (CASPR2), dipeptidyl amino peptidase‐like protein (DPPX) in serum was negative. Paraneoplastic panel was not done. Brain MRI showed abnormal signal, T2/FLAIR (fluid attenuated inversion recovery) sequence showed hyperintensities, T1 sequence showed hypo‐intensities with symmetrical involvement of bilateral medial temporal lobes, bilateral external capsule, basal ganglia, subcortical part of frontal lobes and basifrontal lobes. GRE (Gradient echo) sequences did not show hemorrhages, hence acute hemorrhagic encephalomyelitis which is a form of ADEM was ruled out (Figure 1A–F). Whole spine screening MRI was normal. He was started on injection acyclovir considering the possibility of herpes encephalitis, though herpes PCR was negative. He received injection of mannitol for diffuse cerebral edema. Antiepileptics were continued. EEG was showing few right hemisphere discharges.

There was no improvement with injection acyclovir. Patient was started on injection methylprednisolone on third day of admission as brain MRI was showing diffuse white matter changes which could be due to acute disseminated encephalomyelitis (ADEM) secondary to viral infection. After 5 days of methylprednisolone, patient was started on intravenous immunoglobulins (IVIg), as there was no improvement with steroids. IVIg was given at dose of 0.4 g/kg/day for 5 days. No improvement in his sensorium was seen. He presented a GCS of 5 (E2M2V1). He underwent tracheostomy and percutaneous endoscopic gastrostomy (PEG) during hospital stay. He was discharged after 25 days in altered sensorium with advice to continue nursing care at home. He succumbed to his illness after 2 months.

## 3 DISCUSSION

Acute encephalitis can produce serious disability and significant mortality. The common viruses producing acute encephalitis are herpes group, arboviruses, enteroviruses. Herpes virus produces typical presentation of involvement of temporal lobes, limbic structures, basifrontal cortex. Acute disseminated encephalomyelitis is a known complication after bacterial/viral infection. ADEM is an immune response to different types of infectious agents. Inflammation is supposed to be protective mechanism but during this process, myelin cover is stripped off from nerve fibers. The sequel of this overactive immune reaction can be life‐threatening. This case presented with short duration of fever and altered sensorium. His MRI brain showed extensive confluent lesions extending to all the lobes of cerebrum and basal ganglia. The lesions were not very typical of herpes. The PCR of herpes in CSF came negative twice at a gap of 10 days. The sensitivity and specificity of herpes PCR in CSF is 95% though, it can be negative in early stages of herpes encephalitis. We decided to treat him with injection acyclovir for 3 weeks as he had bilateral temporal lobe involvement. Our patient was given injection methylprednisolone followed by IVIg as we thought that we are dealing with para‐infectious demyelination. ADEM typically shows multifocal asymmetric lesions on MRI.^4^ White matter as well as deep grey matter may be involved. Our case showed more symmetrical, confluent lesions; hence we considered other diagnosis also. There are few other differential diagnoses of radiological changes which are seen in ADEM.^5^ Acute necrotizing encephalitis of childhood (ANEC), mitochondrial encephalopathy, cerebral autosomal dominant arteriopathy with subcortical infarcts and leukoencephalopathy (CADASIL), adult onset leukodystrophy can also cause similar radiological findings. ADEM has been extensively described in pediatric population with similar MRI findings as adults.^6^, ^7^, ^8^ ANEC is mainly seen in children with bilateral symmetrical thalamic lesions. Serum lactate was normal in our case; hence possibility of mitochondrial disease was low. There was no spasticity of limbs or cognitive decline prior to illness, and patient was middle aged, all these findings point against leukodystrophy. The lesions were not typical of Multiple Sclerosis. Our case did not show hemorrhagic foci on gradient echo images of MRI. Hence it was not acute hemorrhagic leukoencephalitis (AHL). AHL, AHEM, and acute necrotizing hemorrhagic leukoencephalitis (ANHLE) of Weston Hurst are different variants of acute, rapidly progressive, and frequently fulminant inflammatory hemorrhagic demyelination of CNS white matter.^8^, ^9^, ^10^, ^11^, ^12^, ^13^, ^14^ Death from brain edema is common within 1 week of onset of encephalopathy. There can be favorable outcomes with early and aggressive treatment with combination of corticosteroids, immunoglobulins, cyclophosphamide, and therapeutic plasma exchange.^10^, ^15^, ^16^, ^17^, ^18^, ^19^ We kept the possibility of extensive encephalitis or ADEM, hence treated with both acyclovir as well as immunotherapy. Our patient did not show improvement with these medications and finally died after 2 months. The in‐hospital mortality of ADEM can be high, ranging from 10 to 20%. Many patients who survive may be left with significant neurological disability. Cost of treatment may be very high in hospital and at home/rehabilitation centers where care is provided. Our case highlights the approach to a patient with febrile illness, seizures and the unusual MRI findings of extensive gray and white matter involvement in brain, which were symmetrical in this case. Use of early immunotherapy may improve outcome. The prognosis in such cases is poor despite optimal medical management.

## 4 CONCLUSIONS

ADEM can be a very fulminant disease. Sometimes radiological findings can be inconclusive. The distinction of viral encephalitis and para infectious demyelination may be blurred. Early immunotherapy is required in fulminant cases.

## AUTHOR CONTRIBUTIONS

**Isha Shukla:** Data curation; writing – original draft; writing – review and editing. **Bhavin Modasia:** Conceptualization; data curation; investigation; visualization. **Bipin Chaurasia:** Supervision; visualization.

## FUNDING INFORMATION

This manuscript did not receive any funds.

## CONFLICT OF INTEREST STATEMENT

The authors have no conflict of interest to declare.

## ETHICS STATEMENT

None.

## CONSENT

Written informed consent was obtained from the patient's next of kin.

## Data Availability

Data sharing not applicable—no new data generated or the article describes entirely theoretical research.
